# Recurrent TransIent Perivascular Inflammation of the Carotid artery syndrome with temporary carotid plaque on ultrasonography: a case report

**DOI:** 10.1002/ccr3.1209

**Published:** 2017-09-26

**Authors:** Akiteru Takamura, Ariyuki Hori

**Affiliations:** ^1^ General Medicine Center Kanazawa Medical University Hospital Uchinada Ishikawa Japan; ^2^ Department of General Medicine Joganji Hospital Toyama Japan

**Keywords:** Carotidynia, temporary carotid plaque, TIPIC syndrome, ultrasonography diagnosis, unilateral neck pain

## Abstract

TransIent Perivascular Inflammation of the Carotid artery (TIPIC) syndrome is a rare syndrome with an unknown cause that is characterized by unilateral neck pain. Its existence as a pathological entity was controversial. We describe a 44‐year‐old male presenting with a 10‐day history of right neck pain diagnosed recurrent TIPIC syndrome with temporary carotid plaque followed by ultrasonography.

## Introduction

TransIent Perivascular Inflammation of the Carotid artery (TIPIC) syndrome is a syndrome with an unknown cause that is characterized by unilateral neck pain that improves spontaneously within a few weeks. It was first reported by Fay in 1927, and it was a controversial diagnosis 1, 2. The International Headache Society Classification Committee criteria for the diagnosis of idiopathic carotidynia indicate that at least one of the following is required overlying the carotid artery: tenderness, swelling, or increased pulsation. Studies have shown that it is a self‐limiting syndrome of <2 weeks and have not demonstrated any structural or anatomical abnormality to be reliably present 3. In addition, Lecler et al. improved the description of an unclassified, clinico‐radiologic entity, which could be described by the proposed acronym: TransIent Perivascular Inflammation of the Carotid artery (TIPIC) syndrome, which has some abnormalities on images in several cases 4. TIPIC syndrome is a diagnosis of exclusion, and the following diagnoses should be excluded before the diagnosis is made: giant cell arteritis, thrombosis, arteriosclerosis, fibromuscular dysplasia, dissection, aneurysm, lymphadenitis, submandibular gland disease, and neck cancer. The existence of the diagnosis is debated, with some researchers insisting that it is not a valid disease, but an inflammatory etiology is supported by the usual response to therapy with nonsteroidal anti‐inflammatory drugs or corticosteroids 5, 6. However, the treatment of TIPIC syndrome remains largely symptomatic and hypothetical, and there have been no placebo‐controlled studies to compare therapies.

## Case Presentation

A 44‐year‐old Japanese male with no significant medical history other than migraines presented to a primary care clinic with 10 days of right neck pain that was not alleviated by treatment with 60 mg of loxoprofen sodium taken three times a day. He had had six episodes of this neck pain in the previous 5 years but had not visited his doctor because it had disappeared within a week each time. He had no history of chronic disease.

On physical examination, the patient was not pale, and his body temperature was 36.6°C. His initial blood pressure was 113/72 mmHg, his heart rate was 72 beats/min, his respiratory rate was 18/min, and his oxygen saturation was 99%. He had severe tenderness over his right carotid bifurcation but had no vascular bruit. The pain was constant and moderate in intensity and occasionally radiated to the inside of his ipsilateral ear and lower jaw. The pain was not made worse by swallowing, but it was exacerbated when he rotated his neck and opened his mouth widely. There was no tenderness over his thyroid or neck muscles. He had no pain elsewhere in his body, and there was no skin rash, lymphadenopathy, or inflammatory changes to his throat. His mucous membranes were moist, heart sounds were normal, lungs were clear, and abdomen was soft and nondistended. He had no peripheral edema.

**Figure 1:**
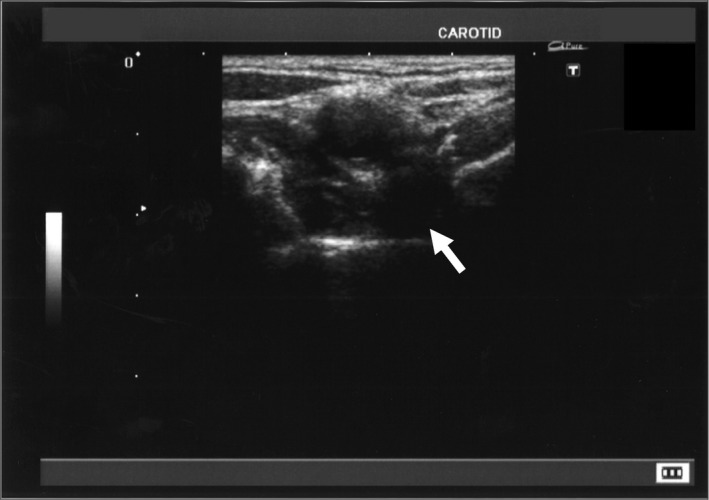
Carotid bifurcation in acute phase; an echo‐poor wall change of outside of carotid artery (white arrow).

**Figure 2:**
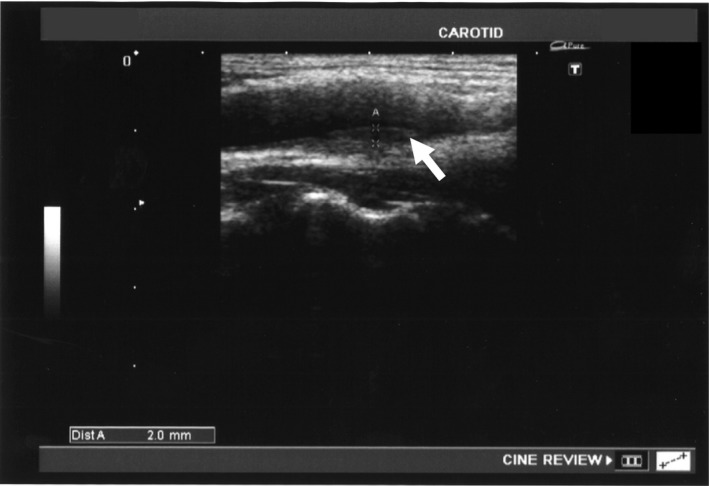
Carotid bifurcation in acute phase; slight outward extension of the vessel (gray arrow) and mild thickening of endothelium slight narrowing of the affected vessel lumen (white arrow).

**Figure 3:**
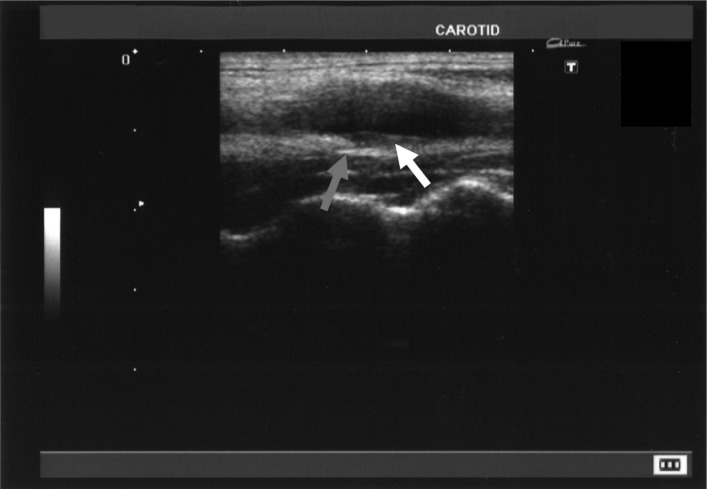
Carotid bifurcation in recovery phase; outward extension of the vessel (gray arrow) and mild thickening of endothelium were being improved (white arrow).

The patient's laboratory values were within normal limits without any inflammatory findings (Table 1). His blood cell count, liver and renal function tests, lipid tests, blood glucose, glycohemoglobin, and electrolytes were also in the normal ranges. There were negative findings for auto‐nuclear antibodies and antineutrophil cytoplasmic antibodies. An ultrasound revealed a hypoechoic change of the outside wall of the carotid artery (Fig. 1), slight outward extension of the vessel, mild thickening of the carotid wall, and mild narrowing of the vessel lumen (Fig. 2). The maximum intima‐media thickness of the right carotid bifurcation decreased in 2 weeks (Fig. 3) over the course of 1 week with the outward extension also disappearing during this time period.

**Table 1:** Laboratory results

Variables	Results	Normal reference range
White blood cells (/*μ*L)	4.3 × 10^3^	4.0–9.0 × 10^3^
Neutrophils (/L)	4.8 × 10^9^	4.0–9.0 × 10^9^
Red blood cells (/*μ*L)	4.9 × 10^7^	4.1–5.6 × 10^7^
Hemoglobin (g/dL)	15.4	12.0–17.0
Platelet (/*μ*L)	22.6	17.0–36.0
TSP (g/dL)	7.4	6.3–8.3
ALB (g/dL)	4.7	3.8–5.3
AST (IU/L)	24	10–40
ALT (IU/L)	21	5–45
CK (IU/L)	58	40–200
T‐Chol (mg/dL)	220	151–254
TG (mg/dl)	102	30–149
HbA1c (%)	5.1	5.4>
BUN (mg/dL)	16	8.6–22.9
CRE (mg/dL)	0.78	0.6–1.1
CRP (mg/dL)	0.03>	0.3>
ESR (mm/1 h)	5	1.0–10.0
ANA (dilutions)	1:40>	1:40>
MPO‐ANCA (dilutions)	1:40>	1:40>
PR3‐ANCA (dilutions)	1:40>	1:40>

TSP, total serum protein; ALB, albumin; AST, aspartate aminotransferase; ALT, alanine aminotransferase; CK, creatine kinase; T‐Chol, total cholesterol; TG, triglyceride; HbA1c, glycohemoglobin; BUN, blood urea nitrogen; CRE, creatinine; CRP, C‐reactive protein; ESR, erythrocyte sedimentation rate; ANA, antinuclear antibody; MPO‐ANCA, myeloperoxidase‐ antineutrophil cytoplasmic antibody; PR3‐ANCA, proteinase‐3‐ antineutrophil cytoplasmic antibody.

Based on the findings described above, we made the diagnosis of TIPIC syndrome. We told the patient the details of this disease, and he consented to our continuing to observe him without any treatment and to follow his condition with ultrasonography. Over the following week, his neck pain improved spontaneously and completely disappeared without any treatment. An ultrasound 2 weeks later showed that the wall bulging, endothelial thickening, and hypoechoic change of the outside of the carotid artery had improved and nearly disappeared.

## Discussion

TransIent Perivascular Inflammation of the Carotid artery syndrome is a syndrome involving unilateral neck pain and associated tenderness over the carotid bifurcation, first described by Fay in 1927 and then by Roseman in 1967 1, 2, 7. The pain of TIPIC syndrome is exacerbated by movement and sometimes by swallowing, chewing, coughing, and yawning. On examination, almost all cases have tenderness over the carotid artery, most commonly at the bifurcation and often along its entire length into the cranium. Laboratory test results, including complete blood count and erythrocyte sedimentation rate, are most commonly normal. The causes of vascular neck pain are extensive and include entities such as dissection, giant cell arteritis, thrombosis, fibromuscular dysplasia, aneurysm, Takayasu arteritis, and nonvascular etiologies such as lymphadenitis and submandibular gland disease 8. After the exclusion of these diseases, TIPIC syndrome should be considered. The neck pain is said usually self‐limited and resolves within 2 weeks with nonsteroidal anti‐inflammatory or steroid medications. The International Headache Society published modified criteria for TIPIC syndrome as carotidynia in 2004, classifying it as a syndrome rather than as a distinct entity 3. In particular, the criteria specify that patients with carotidynia should not have a structural abnormality of the carotid artery. Our patient had been having migraines for the past 20 years. For the last 5 years, he had been having intermittent unilateral neck pain over the right carotid bifurcation. Previous studies have not discussed the frequency of its recurrence and most have not reported abnormal imaging findings in TIPIC syndrome. However, our case and the past studies reported cases have demonstrated one or more of the following abnormal imaging findings: involvement of a contiguous segment of the distal common carotid artery, bulb, or proximal internal carotid artery, mild luminal narrowing, concentric thickening or outward extension of the wall, or fat stranding in the adjacent carotid space. As we did not try other images such as MRI in this time, we cannot discuss the findings of other images 4, 9, 10, 11, 12, 13. Our patient also had abnormal findings on ultrasonography, including a hypoechoic change of the outside wall of the carotid artery and moderate thickening of endothelium with subsequent slight narrowing of the vessel lumen. Our case was unique in terms of the recurrence case of TIPIC syndrome in a young patient without chronic metabolic disease who had fatty plaque that improved at follow‐up imaging. It is not clear why this temporary plaque was in the artery, but it may have been induced by inflammatory changes in the artery wall as the past studies showed, which may help to explain why it resolved with anti‐inflammatory treatment.

## Conclusions

In summary, we described a rare recurrent case of TIPIC syndrome with temporary carotid plaque in young male without any chronic risks followed by ultrasound sonography. In the appropriate clinical context, a diagnosis of TIPIC syndrome as a cause of vascular neck pain may be supported by characteristic radiologic findings. This case report supports the imaging features of TIPIC syndrome previously described in the literature. This disease may easily be missed in primary care settings because of its transient and self‐limiting nature. The patient who has a recurrent history of spontaneously improving neck pain with no positive laboratory findings may likely have TIPIC syndrome, and ultrasound may be useful for diagnosis and follow‐up in patients with unilateral neck pain presenting to their primary care physicians.

## Authorship

AT and AH: contributed equally to the patient's care. AT: wrote the manuscript. Both authors read and approved the final manuscript.

## Ethics Approval and Consent to Participate

This report was approved by the Joganji Hospital Ethics Committee.

## Consent for Publication

Written informed consent was obtained from the patient for publication of this case report and the accompanying images. A record of the consent in the medical record is available for review by the editor‐in‐chief of this journal.

## Conflict of Interest

The authors declare that they have no competing interests.
